# Survival outcomes with warfarin compared with direct oral anticoagulants in cancer-associated venous thromboembolism in the United States: A population-based cohort study

**DOI:** 10.1371/journal.pmed.1004012

**Published:** 2022-05-25

**Authors:** Adeel M. Khan, Thita Chiasakul, Robert Redd, Rushad Patell, Ellen P. McCarthy, Donna Neuberg, Jeffrey I. Zwicker

**Affiliations:** 1 Division of Hematology and Division of Hemostasis and Thrombosis, Department of Medicine, Beth Israel Deaconess Medical Center, Harvard Medical School, Boston, Massachusetts, United States of America; 2 Division of Hematology, Department of Medicine, Faculty of Medicine, Chulalongkorn University and King Chulalongkorn Memorial Hospital, Thai Red Cross Society, Bangkok, Thailand; 3 Department of Data Science, Dana-Farber Cancer Institute, Boston, Massachusetts, United States of America; 4 Hinda and Arthur Marcus Institute for Aging Research, Hebrew SeniorLife, Boston, Massachusetts, United States of America; 5 Divisions of Gerontology and General Medicine, Department of Medicine, Beth Israel Deaconess Medical Center, Harvard Medical School, Boston, Massachusetts, United States of America; Leiden University Medical Center, NETHERLANDS

## Abstract

**Background:**

Direct oral anticoagulants (DOACs) have comparable efficacy with low-molecular-weight heparin (LMWH) for the treatment of cancer-associated venous thromboembolism (VTE). Whether there is a mortality benefit of DOACs compared with warfarin in the management of VTE in cancer is not established.

**Methods and findings:**

Utilizing the United States’ Surveillance, Epidemiology, and End Results (SEER)-Medicare linked databases from 2012 through 2016, we analyzed overall survival in individuals diagnosed with a primary gastric, colorectal, pancreas, lung, ovarian, or brain cancer and VTE who received a prescription of DOAC or warfarin within 30 days of VTE diagnosis. Patients were matched 1:2 (DOAC to warfarin) through exact matching for cancer stage and propensity score matching for age, cancer site, cancer stage, and time interval from cancer to VTE diagnosis. The analysis identified 4,274 patients who received a DOAC or warfarin for the treatment of VTE within 30 days of cancer diagnosis (1,348 in DOAC group and 2,926 in warfarin group). Patients were of median age 75 years and 56% female. Within the DOAC group, 1,188 (88%) received rivaroxaban, and 160 (12%) received apixaban. With a median follow-up of 41 months, warfarin was associated with a statistically significantly higher overall survival compared to DOACs (median overall survival 12.0 months [95% confidence interval (CI): 10.9 to 13.5] versus 9.9 months [95% CI: 8.4 to 11.2]; hazard ratio (HR) 0.85; 95% CI: 0.78 to 0.91; *p* < 0.001). Observed differences in survival were consistent across subgroups of cancer sites, cancer stages, and type of VTE. The study limitations include retrospective design with potential for unaccounted confounders along with issues of generalizability beyond the cancer diagnoses studied.

**Conclusions:**

In this analysis of a population-based registry, warfarin was associated with prolonged overall survival compared to DOACs for treatment of cancer-associated VTE.

## Abstract 2

Adeel Khan and co-workers study anticoagulant prescriptions and survival outcomes in patients with cancer-associated venous thromboembolism.

## Author summary

**Why was this study done?:**

- In population-based studies, warfarin has been associated with improved overall survival compared with low-molecular-weight heparin (LMWH) for the treatment of cancer-associated thrombosis.
- Based on demonstration of antithrombotic efficacy, direct oral anticoagulants (DOACs) are commonly prescribed for management of venous thromboembolism (VTE) in cancer.
- Whether there is an overall survival difference in the treatment of VTE in cancer with DOACs compared with warfarin is not known.

**What did the researchers do and find?:**

- This was a retrospective cohort study of the Surveillance, Epidemiology, and End Results (SEER)-Medicare database comparing the overall survival of exact-stage and propensity score–matched elderly patients with cancer and VTE (*N* = 4,274) who received DOACs or warfarin.
- Warfarin was associated with a significantly prolonged overall survival compared with DOACS in the treatment of VTE in cancer.

**What do these findings mean?:**

- Emerging population-based evidence suggests an overall survival benefit with warfarin compared with other anticoagulants for treatment of VTE in cancer.
- Warfarin has been shown to have antineoplastic activity in preclinical models.
- Prospective randomized trials evaluating potential survival differences with warfarin are warranted.

## Introduction

Cancer-associated thromboembolism (CAT) is a common cause of mortality in patients with malignancy [1]. Approximately 20% of patients with a known cancer experience venous thromboembolism (VTE) [2]. Different anticoagulants have been evaluated for the management of thrombosis in cancer. Low-molecular-weight heparin (LMWH) demonstrated superior efficacy in reducing recurrent VTE when compared with the previous standard of warfarin at 6 months [3]. More recently, direct oral anticoagulants (DOACs) demonstrated similar, if not improved, outcomes when compared with LMWH [4–7]. A survival benefit for one anticoagulant over another has not been demonstrated in randomized trials, although these trials included a wide range of cancer diagnosis, across different stages and durations of cancer diagnosis, and were of limited treatment duration (most commonly 6 months) [8].

As the survival benefit of different anticoagulants based on a reduction of fatal pulmonary emboli is likely to be modest, we speculated that a large cohort followed for an extended period of time would be required. Accordingly, we previously evaluated whether the demonstrated anticoagulant superiority of LMWH would improve survival outcomes compared with warfarin for the treatment of CAT [9]. Surprisingly, warfarin resulted in a clear survival benefit compared to LMWH (HR 0.86, 95% confidence interval (CI): 0.83 to 0.90; *p* < 0.001). The benefit of warfarin was observed across various subgroups including different cancer types, stages, age, comorbidity burden, and year of VTE.

These results are not without precedent as a Finnish study that included over 6,000 men with cancer, the use of warfarin was associated with improved survival relative to other anticoagulants [10]. Another population-based study demonstrated a significantly lower incidence of cancer among individuals receiving warfarin compared with nonusers (incidence rate ratio 0.84, 95% CI: 0.82 to 0.86) [11]. These results are provocative and suggest that warfarin may provide survival benefit independent of anticoagulation effectiveness as has been demonstrated in preclinical models [12–18]. A potential confounder that we were not able to address in the prior investigation of warfarin versus LMWH was whether the observed survival differences were due to selection bias of healthier individuals who were able to tolerate oral medications and thus had an intrinsically better prognosis relative to LMWH [9]. In the present study, all patients received oral anticoagulation, i.e., warfarin or a DOAC.

Based on their ease-of-use along with favorable efficacy and safety profiles, DOACs are quickly becoming standard-of-care for the management of VTE in cancer patients [19]. We evaluated whether there was a survival difference among cancer patients treated for VTE with either warfarin or DAOCs in a population-based database.

## Methods

### Study design

This is a retrospective cohort of individuals diagnosed with primary gastric, colorectal, pancreatic, lung, ovarian, or brain cancer from January 1, 2012 to December 31, 2015 in the Surveillance, Epidemiology, and End Results (SEER) Registries linked to Medicare enrollment data and claims through December 31, 2016 (which was the latest available dataset at time of analysis) [20]. Although a written analysis plan is not available, this planned, prespecified analysis mirrored the published analyses comparing survival outcomes in warfarin versus LMWH [9]. The study received institutional review board approval at Beth Israel Deaconess Medical Center. All data were deidentified from the SEER-Medicare database.

### Data source

All data were obtained from the SEER-Medicare database. This is a national registry program that provides individual-level linkage of SEER cancer registry data with Medicare enrollment and claims data. We utilized SEER-18, which contains 18 registry sites across the US. In total, its geographic coverage entails 28% of the US population [20]. Medicare is a federal health insurance system under the Centers for Medicare & Medicaid Services that encompasses 94% of persons aged 65 years or older in the US. Medicare data contain beneficiaries’ enrollment (namely Parts A, B, C, and D), claims for inpatient, provider, and outpatient services among fee-for-service beneficiaries as well as prescription drug claims for beneficiaries with Part D coverage. We utilized Medicare claims data through September 30, 2015 with follow-up for survival data through December 31, 2016.

### Study cohort

Patients were included in the present study if they met all of the following criteria: diagnosis of primary gastric, colorectal, pancreatic, lung, ovarian, or brain cancer between 2012 and 2016; an index VTE event was either contemporaneous with cancer diagnosis (within 1 month) or at any time after establishment of the diagnosis; were aged 66 years or older at the time of VTE event; had available prescription claims data for apixaban, rivaroxaban, edoxaban, dabigatran, or warfarin within 30 days; and survived at least 14 days after the index VTE event. These 6 solid tumors were selected due to their relatively high rates of VTE. Patients were excluded from the study population if they were originally entitled to Medicare before the age of 65 due to disability or end-stage renal disease and were not enrolled in fee-for-service Medicare Parts A, B, and D at the time of VTE diagnosis. The index VTE event was determined by Medicare claims (either inpatient or provider/outpatient) that contained a previously validated set of International Classification of Diseases, 9th Revision, Clinical Modification (ICD-9-CM) diagnosis codes for VTE in any diagnosis position (**S1 Table**) [21]. DOACs (i.e., apixaban, rivaroxaban, dabigatran, and edoxaban) and warfarin prescriptions were identified in Medicare Part D data using National Drug Codes.

Eligible patients were assigned to the DOAC group or warfarin group based on the first anticoagulant prescription within 30 days after the index VTE event. To account for bridging treatment to attain therapeutic levels of warfarin, patients whose first anticoagulant prescription was LMWH, heparin, or another non-DOAC but received a warfarin prescription within 14 days were classified as part of the warfarin group. Similarly, patients whose first anticoagulant prescription was LMWH, heparin, or another non-warfarin anticoagulant but received a DOAC prescription within 14 days were classified as part of the DOAC group. Patients receiving anticoagulants for other indications (i.e., atrial fibrillation) prior index VTE qualification were not excluded. To address the potential for immortal time bias related to the warfarin prescription, inclusion in both treatment groups required that patients survived at least 14 days following the index VTE.

### Variables of interest

Included variables pertained to the patient’s demographics (age at VTE diagnosis, sex, and race), cancer (primary site, stage, year of diagnosis, and active anticancer therapy), and index VTE (year of diagnosis, type of VTE, and time from cancer diagnosis to index VTE). Patients’ socioeconomic status was assessed by median income, poverty level, and education level of the census tract of residence.

To assess comorbidity burden, the Elixhauser Comorbidity Index (range 0 to 31) was calculated for hospitalized patients only. This index was developed and validated using information derived from hospitalizations and includes conditions such as presence of heart failure, chronic pulmonary disease, diabetes, and other acute and chronic illnesses. The Elixhauser index was selected over the Charlson index due to data showing superior association with mortality [22–24]. The score was not calculated for nonhospitalized patients as they did not have the requisite data to compute an Elixhauser comorbidity index.

Stage at diagnosis was defined according to the criteria set by American Joint Committee on Cancer, 7th edition. Lung cancer subtype was identified using the International Classification of Diseases for Oncology, 3rd Edition (ICD-O-3) histology codes (**S2 Table**). Systemic anticancer therapies were identified using ICD-9-CM diagnosis and procedure codes, Healthcare Common Procedure Coding System (HCPCS) codes, and National Drug Codes related to chemotherapy administration and prescription of approved drugs for included cancer types [25,26]. Antineoplastic qualification was broadly inclusive of cytotoxic, tyrosine kinase inhibitors, and immunotherapy (complete coding available upon request). Patients were considered to have received active systemic anticancer therapy if they had at least 1 claim (inpatient, outpatient, carrier/provider, Durable Medical Equipment, and Part D Event) with a corresponding therapy code within the 3 months preceding and after the diagnosis of index VTE.

### Outcomes

The primary outcome of interest was overall survival defined as the time from the diagnosis of the index VTE event to death from any cause at the time of data cutoff of December 31, 2016. The date of VTE diagnosis was determined using the date of admission (for patients diagnosed in a hospital) or the date of service (for patients diagnosed in outpatient settings). Dates of death and last follow-up were extracted from the Medicare Beneficiary Summary file.

### Statistical analysis

To minimize imbalances between the DOAC group and warfarin group, a propensity score matching algorithm was used to equalize patients on certain variables such as cancer diagnosis, cancer stage, age, year of VTE, and length of time from cancer diagnosis until study entry [27]. Patients were exact-matched for cancer stage (as stage 1 to 2, stage 3, stage 4, not applicable, and unknown stage) and propensity score–matched based on age (<75 years versus ≥75 years), primary cancer site, year of VTE diagnosis, and time from cancer diagnosis to index VTE (within 1 month or up to 3 months after versus more than 3 months after cancer diagnosis). Patients in the DOAC group were matched to patients in the warfarin group using 1:2 nearest neighbor matching without replacement.

Survival distributions were estimated by the method of Kaplan and Meier. The median overall survival and 95% CIs were reported with Greenwood’s formula for estimated variance. The overall mortality rate at 90 days by treatment group was compared using Fisher exact test. Cox proportional hazards models were used to estimate the hazard ratio (HR) for death with 95% CIs. Prespecified subgroup analyses of overall survival were performed based on age at index VTE diagnosis, sex, race, Elixhauser Comorbidity Index (only for hospitalized patients as only these patients had requisite claims data for calculation), primary cancer site, lung cancer histologic subtype (small cell versus non-small cell), cancer stage (for all non-brain cancers and non-small-cell lung cancer), type of VTE (deep vein thrombosis versus pulmonary embolism), and setting of VTE (inpatient versus outpatient). No adjustment for multiple comparisons was planned for these subgroup analyses to minimize the potential for type II errors. Sensitivity analyses of the primary outcome were performed with the exclusion of pancreatic cancer to explore the robustness of the results. All reported *p*-values are 2 sided, and all the analyses were performed with the use of SAS software, version 9.4 (SAS Institute) and R version 4.0.2 (R Foundation for Statistical Computing). A STrengthening the Reporting of OBservational studies in Epidemiology (STROBE) checklist was completed (see S1 STROBE Checklist).

## Results

### Study cohort

A total of 4,274 eligible patients were identified from 2012 to 2016. After propensity score matching, 4,044 were included in the final analysis: 1,348 in the DOAC group (having received apixaban or rivaroxaban) and 2,696 in the warfarin group (**S1 Fig**). The baseline characteristics of the matched cohort are presented in **Table 1** (see also **S3 Table**). Overall, 1,188 (88%) received rivaroxaban, and 160 (12%) received apixaban in the DOAC group. In both cohorts, the median age at the time of index VTE was 75 years, and 56% were female. Socioeconomic characteristics were similar between the DOAC and warfarin groups (**S4 Table**).

**Table 1:** Demographic and clinical characteristics of study cohort at baseline after propensity score matching.

Characteristics	Total (*N* = 4,044)	Warfarin (*N* = 2,696)	DOAC (*N* = 1,348)
Age at index VTE diagnosis			
Median (IQR)—yr	75 (70 to 81)	75 (70 to 81)	75 (70 to 81)
66 to 74 yr—no. (%)	1,892 (47)	1,248 (46)	644 (48)
≥75 yr—no. (%)	2,152 (53)	1,448 (54)	704 (52)
Female sex—no. (%)	2,284 (56)	1,523 (56)	761 (56)
Race—no. (%)*			
White	3,353 (83)	2,206 (82)	1,147 (85)
Black	430 (11)	299 (11)	131 (10)
Other or unknown	261 (6)	191 (7)	70 (5)
Median Elixhauser Comorbidity Index score, Median (IQR)	5 (4 to 7)	5 (4 to 7)	5 (4 to 7)
Cancer stage at diagnosis—no. (%)^‡^			
Stage 1 to 2	1,170 (29)	780 (29)	390 (29)
Stage 3	1,116 (28)	744 (28)	372 (28)
Stage 4	1,371 (34)	914 (34)	457 (34)
Not applicable	189 (5)	126 (5)	63 (5)
Unknown	198 (5)	132 (5)	66 (5)
Primary cancer site—no. (%)			
Gastric	172 (4)	113 (4)	59 (4)
Colorectal	1,376 (34)	930 (34)	446 (33)
Pancreatic	532 (13)	325 (12)	207 (15)
Lung	1,624 (40)	1,066 (40)	558 (41)
Ovarian	199 (5)	168 (6)	31 (2)
Brain	141 (3)	94 (3)	47 (3)
Systemic anticancer therapy within 3 months—no. (%)	1,522 (38)	963 (36)	559 (41)
Year of index VTE diagnosis			
2012	904 (22)	883 (33)	21 (2)
2013	1,065 (26)	767 (28)	298 (22)
2014	1,186 (29)	634 (24)	552 (41)
2015	889 (22)	412 (15)	477 (35)
Type of index VTE			
Deep vein thrombosis	2,349 (58)	1,561 (58)	788 (58)
Pulmonary embolism	1,695 (42)	1,135 (42)	560 (42)
Time from cancer diagnosis to index VTE			
Median months	4.8	4.9	4.6
(IQR)	(1.1 to 20.6)	(1.1 to 21.0)	(1.1 to 19.5)

* Race was abstracted from the Medicare Beneficiary Summary File. Other included Other, Asian, Hispanic, and North American Native.

† The Elixhauser Comorbidity Index scores range from 0 to 31, with higher scores indicating a higher number of chronic coexisting conditions. The scores were calculated only for inpatient claims.

‡ Based on the staging criteria of American Joint Committee on Cancer, 7th edition.

DOAC, direct oral anticoagulant; IQR, interquartile range; VTE, venous thromboembolism.

Among the hospitalized patients (*n* = 2,102 [52%]), comorbidity burden was similar between both groups with an Elixhauser index of 5 (IQR 4 to 7). Median time from cancer diagnosis to the VTE event was 4.6 months (IQR 1.1 to 19.5) in the DOACs group and 4.9 months (IQR 1.1 to 21.0) in the warfarin group. In both groups, there was a greater percentage of deep vein thrombosis (58%) than pulmonary embolism (42%) as the index VTE event.

The most common malignancies were lung cancer (40.2%), colorectal cancer (34.0%), and pancreatic cancer (13.2%). Cancer types were balanced across the DOAC and warfarin groups in all primary sites except for a greater proportion of ovarian cancer in the warfarin group (6%) compared to the DOAC group (2%) and greater proportion of pancreatic cancer in the DOACs group (15%) compared to the warfarin group (12%). A plurality of patients had stage 4 disease (34%) in both groups followed by similar proportions of stage 1 to 2 (29%) and stage 3 (28%) disease. There was a higher proportion of patients who received systemic anticancer therapy within 3 months of the VTE diagnosis in the DOAC group (41%) compared to the warfarin group (36%).

### Overall survival

**Fig 1:**
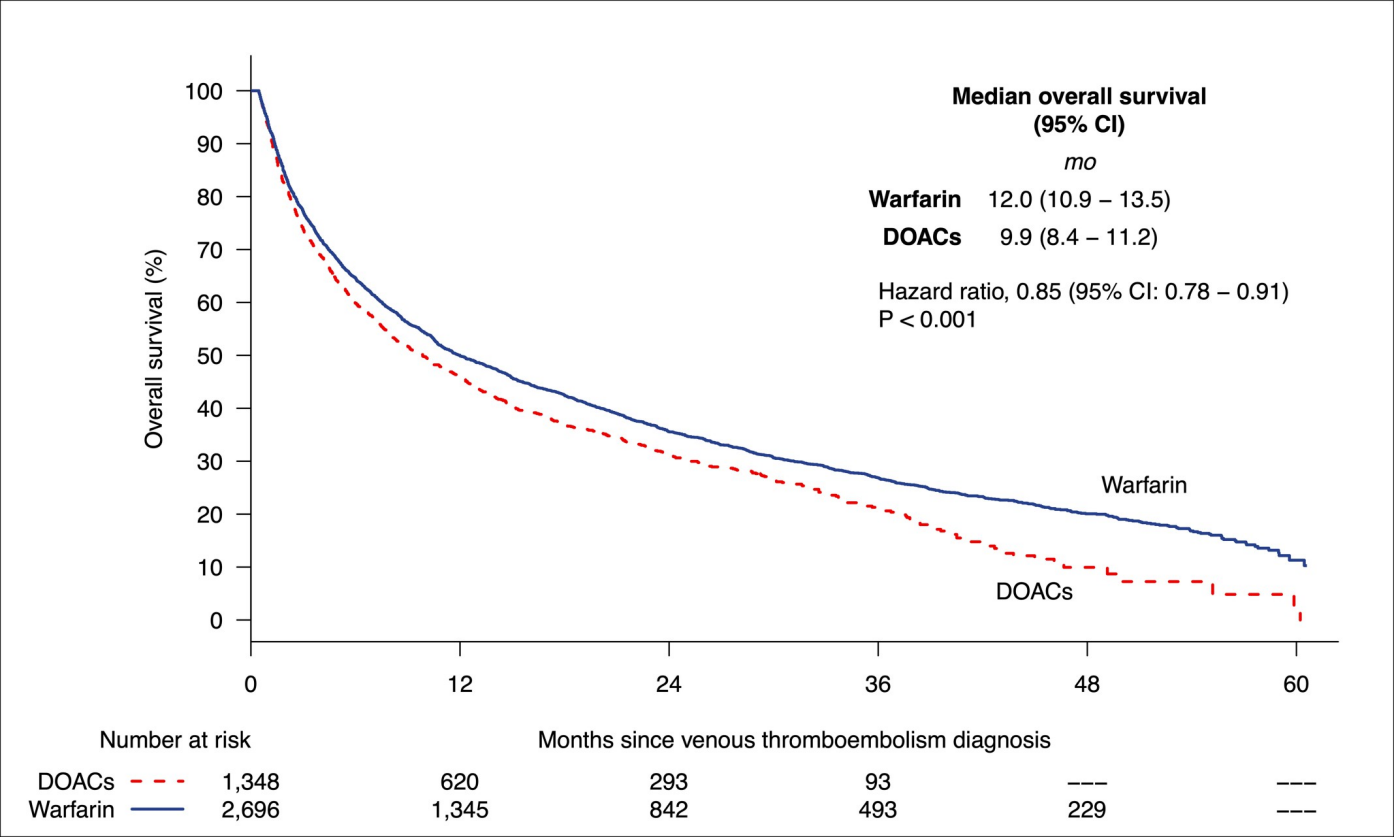
Kaplan–Meier curves of overall survival in DOAC-treated patients (dashed line) versus warfarin-treated patients (solid line) with cancer-associated VTE. CI, confidence interval; DOAC, direct oral anticoagulant; VTE, venous thromboembolism.

The median follow-up was 41 months (range 0 to 60). Warfarin was associated with significantly improved overall survival compared to DOACs (median overall survival 12.0 months [95% CI: 10.9 to 13.5] versus 9.9 months [95% CI: 8.4 to 11.2]). HR for death was 0.85 (95% CI: 0.78 to 0.91; *p* < 0.001) for the warfarin group compared to the DOAC group (**Fig 1**). At 90 days, overall mortality rate was 23% in warfarin group and 26% in the DOAC group *(p <* 0.001).

### Overall survival by cancer stages

**Fig 2:**
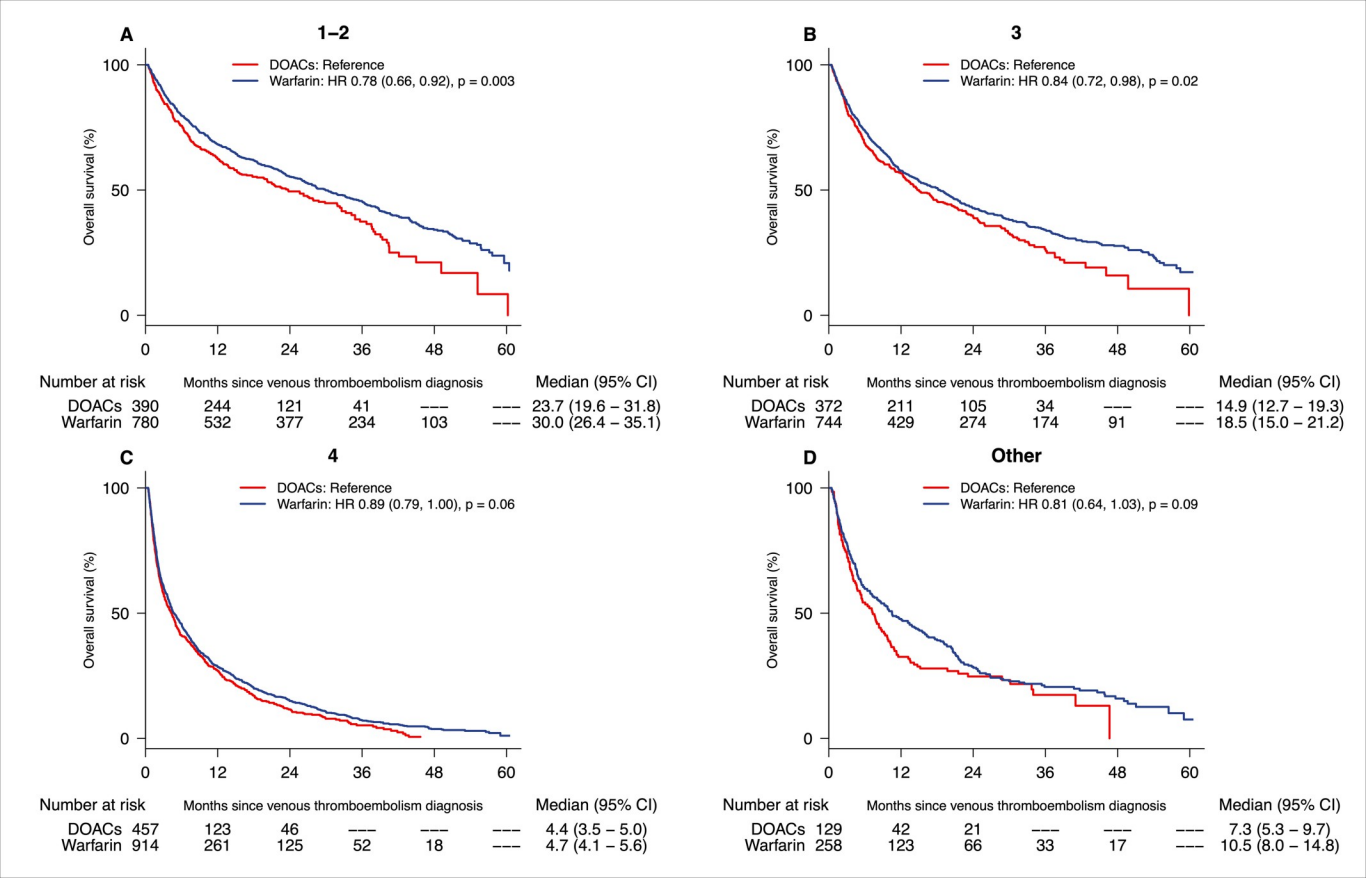
Kaplan–Meier estimate of overall survival with DOAC-treated patients (red line) compared to warfarin (blue line) in all cancers with stages 1 to 2 (panel A), stage 3 (panel B), stage 4 (panel C), and stage not applicable and unknown (panel D). CI, confidence interval; DOAC, direct oral anticoagulant; HR, hazard ratio.

Kaplan–Meier curves for overall survival by cancer stage (stage 1 to 2, 3, 4, stage not applicable, and stage unknown) are shown in **Fig 2**. Warfarin was associated with improved overall survival compared with DOACs across different cancer stages. This difference was most evident in the earlier stage group (stage 1 to 2) with median overall survival of 30.0 months (95% CI: 26.4 to 35.1 months) in the warfarin group compared to 23.7 months (95% CI: 19.6 to 31.8 months) in the DOAC group, yielding a highly significant HR of 1.24 (95% CI: 1.06 to 1.45, *p* = 0.009). Similarly, the stage 3 cohort, median survival was increased by 3.6 months (HR 1.18, 95% CI: 1.02 to 1.38). In the stage 4 group, the median overall survival was 4.7 months (95% CI: 4.1 to 5.6 months) in the warfarin group versus 4.4 months (95% CI: 3.5 to 5.0 months) in the DOACs group (HR 1.12, 95% CI: 0.99 to 1.25, *p* = 0.06).

### Overall survival by subgroups

**Fig 3:**
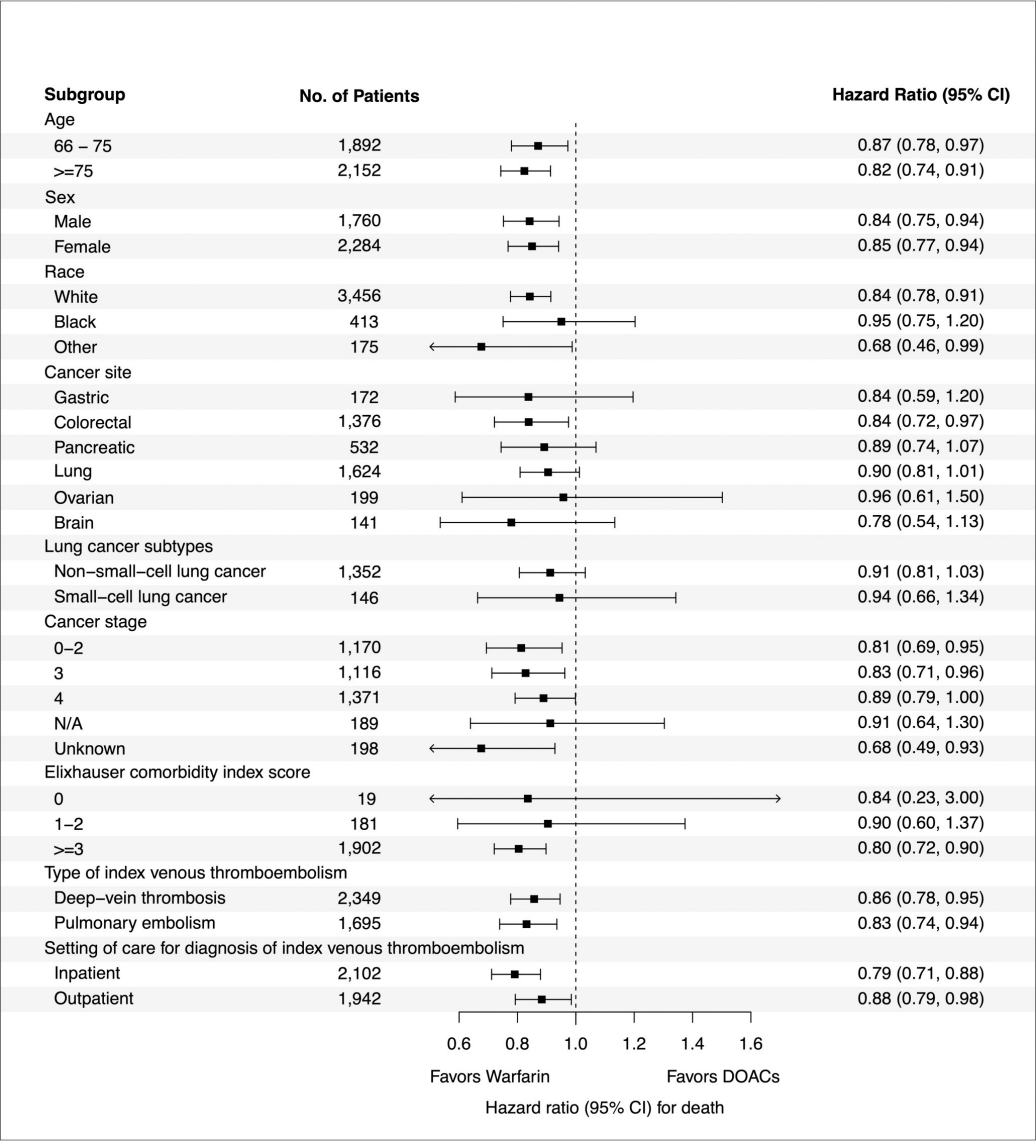
Forest plot of overall survival for subgroup analyses using a multivariable Cox proportional hazards model that included the anticoagulant group, the subgroup covariate of interest, and the subgroup-by-treatment interaction. CI, confidence interval; DOAC, direct oral anticoagulant.

We analyzed survival differences for warfarin versus DOACs across a number of subgroups (**Fig 3**). In all instances, improved survival appeared to favor warfarin over DOACs. Accordingly, warfarin was associated with a significant survival benefit that was independent of age, sex, receipt of systemic anticancer therapy, type of incident VTE (i.e., pulmonary embolism or deep venous thrombosis), and inpatient or outpatient management. Improved overall survival was most evident in nonmetastatic disease. Due to limited sample size for individual tumor types, we cannot definitively conclude survival differences for specific cancer subtypes. The most definitive survival benefit observed for an individual malignancy was colorectal cancer (HR 0.85, 95% CI: 0.72 to 0.97). In a post hoc analysis, we also assessed survival differences among those who received at least 3 months of anticoagulation. A total of 64% of patients receiving warfarin and 57% of patients receiving a DOAC completed at least 3 months of anticoagulation. In a landmark analysis for those who received at least 3 months of anticoagulation, the median survival with warfarin was 23.3 months compared with 21.6 months with DOACs (HR 0.86, 95% CI: 0.77 to 0.96, *p* = 0.006).

## Discussion

DOACs have similar efficacy and potentially less bleeding when compared with warfarin for the treatment of acute VTE in the noncancer population [28,29]. Because of the established superiority in preventing recurrent VTE in cancer populations, all comparative clinical trials in cancer to date with DOACs have included LMWH as the active comparator rather than warfarin [4–7,30]. DOACs are now commonly used for the treatment of cancer-associated thrombosis but whether there is a mortality difference relative to warfarin is not known. In light of the emerging evidence that warfarin is associated with improved survival in cancer patients, we performed this analysis of survival outcomes in cancer patients who received warfarin or a DOAC in the management of acute VTE [9]. In this population-based linked analysis of over 4,200 patients with cancer-associated thrombosis, warfarin was associated with improved overall survival compared with apixaban and rivaroxaban for treatment of VTE. This association was consistent across different subgroups including primary cancer, stage, Elixhauser comorbidity index, age, sex, type of VTE, and time of VTE.

Warfarin has been linked with improved overall survival in patients with cancer. Several large Finnish population–based studies identified an improved survival in different cancer cohorts with warfarin compared with non-warfarin anticoagulation for all indications (VTE and atrial fibrillation) [10,31,32]. Our prior study using the SEER-Medicare database involving over 9,700 patients showed that warfarin was associated with superior survival compared with LMWH for the treatment of cancer-associated VTE [9]. Analyses of survival outcomes in atrial fibrillation clinical trials appear to support these findings. For instance, in the Aristotle trial, among the 1,236 participants with a history of cancer or active cancer, there were 54 deaths among those receiving apixaban compared with 42 deaths in the warfarin group (HR 1.32, 95% CI: 0.88 to 1.97) [33]. The study was underpowered to draw conclusions regarding impact on mortality among the relatively few patients enrolled with active cancer, but among the 1,079 patients with a history of cancer, the use of warfarin was associated with significantly improved overall survival compared with apixaban (HR 0.61, 95% CI: 0.39 to 0.96). Similarly, among the patients with malignancy followed on the ENGAGE TF-TIMI 48 Trial, all-cause death was 12.4% among 390 patients with cancer randomized to edoxaban 60 mg daily compared with 11.5% among 395 patients randomized to warfarin (HR 1.09, 95% CI: 1.09 to 1.41) [34]. Confounding potential antineoplastic mortality signal in these atrial fibrillation trials was the significant advantage in reducing ischemic endpoints with DOAC compared with warfarin [33–34].

We acknowledge several potential limitations of the study and considered numerous potential biases and sources of confounding in the conduct of our analyses. To minimize imbalances between the groups, we used propensity score matching including exact-matching for cancer stage. The groups were balanced for age, sex, race, Elixhauser comborbidity index, type of index VT, and socioeconomic status (e.g., marital status, education, and income). The widespread use of DOACs began in 2013 such that there were comparably more patients receiving warfarin toward the beginning of study period (2012 through 2015). The median time to cancer diagnosis to VTE was also slightly longer in the warfarin arm, which could potentially bias the findings against the warfarin group considering the survival comparisons were relative to diagnosis of VTE. The databases do not permit analysis of warfarin anticoagulation efficiency so we cannot conclude whether degree of anticoagulation was associated with improved outcomes. To assess the small imbalance in pancreatic cancer (12% in warfarin and 15% in DOAC groups), we performed a sensitivity analysis excluding this diagnosis and results remained consistent. In addition, although the higher proportion of patients who received systemic anticancer therapy within 3 months of the VTE diagnosis in the DOAC group (41% versus 36%) may have influenced survival, subgroup analyses demonstrated consistent outcomes regardless of systemic anticancer therapy status. Despite our attempts to minimize bias, we recognize that we cannot exclude the influence of unaccounted confounders. However, these data provide reassurance that the previously observed association of improved survival with warfarin versus LMWH was not due to simply selecting a population with intrinsically better outcomes because they were able to tolerate oral medications.

We also considered the potential for misclassification of VTE and utilized validated sets of diagnostic codes previously published for index VTE with a positive predictive value of 95% for identifying acute VTE [21]. Acknowledging that the positive predictive value is lower for diagnostic codes in the secondary position, we implemented confirmation of VTE by mandating anticoagulant prescription to be within 30 days of the VTE diagnosis [35]. Similarly, we sought to account the potential for immortal time bias in that warfarin prescription is often delayed after a period of bridging anticoagulation. To account for excess early deaths occurring in this window prior to warfarin prescription, study inclusion required patients to survive at least 14 days after following the initial VTE diagnosis.

The SEER-Medicare database primarily includes older adults (more than 65 years of age), thereby precluding the generalizability of our findings to younger cancer populations. Nonetheless, the survival HRs for warfarin versus DOACs were similar for patients 66 to 75 years (HR = 0.87, 95% CI: 0.78 to 0.97) and patients ≥ 75 years (HR = 0.82, 95% CI: 0.74 to 0.91). We note that survival benefit was most pronounced in the group with earlier stage malignancies. This finding was consistent with prior studies and suggest that warfarin may have antimetastatic activity [12–18].

Similarly, although this analysis only allows for a correlative association due to its nonrandomized retrospective nature, possible causal explanations exist based on prior biological work. In preclinical models, warfarin has demonstrated antineoplastic effects [12–18,36,37]. Warfarin has been shown to block the g-carboxylation growth arrest-specific 6 (GAS6) protein, which decreases tumor cell migration and proliferation [37]. In the 1980s, there were trials specifically conducted to evaluate the potential antineoplastic activity of warfarin. This included the US Veterans Administration Cooperative Study-75, which enrolled 431 patients with lung, colon, head and neck, and prostate cancer of different stages and histologies (including both non-small cell and small cell lung cancer) [38]. There was not an observed survival advantage across all groups but survival was doubled in those with small-cell lung cancer *(p* = 0.018). In the Cancer and Leukemia Group B (CALGB) trial, the combination of sub-therapeutic warfarin (international normalized ratio target of 1.6 to 1.9) with chemotherapy and radiation was associated with a nonsignificant improvement in survival among patients with small-cell lung cancer (*p* = 0.07) [39]. The majority of these studies enrolled advanced stage disease, whereas the greatest survival benefit we observed was in earlier stages [9]. Accordingly, in a study that included 800 patients with acute VTE, the use of warfarin reduced incidence of cancer diagnosis by 35% [40].

Several recent randomized studies have established the safety and efficacy of DOAC for the management of acute VTE in patients with cancer. The results of this retrospective analysis using national data from the US show superior survival of warfarin compared to DOACs. The question emerging is not whether DOAC or LMWH are more effective in reducing recurrent VTE but whether warfarin portends a survival benefit in cancer patients that extends beyond its anticoagulant efficacy.

## Supporting information

S1 STROBE Checklist Completed checklist of the observational study. STROBE, STrengthening the Reporting of OBservational studies in Epidemiology.
(DOCX)

S1 Fig Participant disposition in study cohort selection. (DOCX)

S1 Table ICD-9-CM diagnosis codes for VTE. ICD-9-CM, International Classification of Diseases, 9th Revision, Clinical Modification; VTE, venous thromboembolism.
(DOCX)

S2 Table ICD-O-3 histology codes for lung cancer subtypes. ICD-O-3, International Classification of Diseases for Oncology, 3rd Edition.
(DOCX)

S3 Table Pre- and post-matched characteristics of study cohort. (DOCX)

S4 Table Socioeconomic characteristics of study cohort. (DOCX)

## Abbreviations:

CALGB: Cancer and Leukemia Group B
CAT: cancer-associated thromboembolism
CI: confidence interval
DOAC: direct oral anticoagulant
GAS6: growth arrest-specific 6
HCPCS: Healthcare Common Procedure Coding System
ICD-O-3: International Classification of Diseases for Oncology, 3rd Edition
ICD-9-CM: International Classification of Diseases, 9th Revision, Clinical Modification
LMWH: low-molecular-weight heparin
SEER: Surveillance, Epidemiology, and End Results
STROBE: STrengthening the Reporting of OBservational studies in Epidemiology
VTE: venous thromboembolism
