# Supplementary material for: Survival outcomes with warfarin compared with direct oral anticoagulants in cancer-associated venous thromboembolism in the United States: A population-based cohort study
Source: PLoS Med. 2022 May 25;19(5):e1004012. doi: 10.1371/journal.pmed.1004012 (PMC9182592; doi:10.1371/journal.pmed.1004012)
Supplement: S1 Fig — (DOCX) [file pmed.1004012.s002.docx]

**Supplemental Figure 1.** Participant Disposition in Study Cohort Selection.

754,469 Patients were diagnosed with gastric, colorectal, pancreatic, lung, ovarian or brain cancer in between 2007 – 2015

1,348 Were included in the DOACs group

2,696 Were included in the warfarin group

49,337 Were diagnosed with eligible index venous thromboembolism

705,132 Were excluded

153,008 Were not entitled to Medicare by old age

502,260 Did not have VTE diagnosis during observation period

15,790 Had VTE diagnosis that preceded cancer diagnosis by more than 6 months

3,848 Were <66 years at index VTE diagnosis

30,226 Were not enrolled in Medicare Part A, B or D at the time of VTE diagnosis

4,044 Were included in the propensity-score matched analysis

4,268 Were excluded

3,444 did not have VTE 2012-2015

670 had VTE diagnosed more than 1 month before cancer diagnosis

81 Did not survive 14 days after VTE

73 Had cancer stage 0

637 Were not included based on lack of acceptable propensity-score matching

277 Received DOACs

360 Received Warfarin

40,388 Were excluded

34,549 Did not have anticoagulant prescription within 30 days after diagnosis

5,839 Received LMWH

8,949 Received DOACs or Warfarin within 30 days after diagnosis of venous thromboembolism
