# Supplementary material for: Survival outcomes with warfarin compared with direct oral anticoagulants in cancer-associated venous thromboembolism in the United States: A population-based cohort study
Source: PLoS Med. 2022 May 25;19(5):e1004012. doi: 10.1371/journal.pmed.1004012 (PMC9182592; doi:10.1371/journal.pmed.1004012)
Supplement: S1 Table — ICD-9-CM, International Classification of Diseases, 9th Revision, Clinical Modification; VTE, venous thromboembolism. (DOCX) [file pmed.1004012.s003.docx]

**Supplemental Table 1.** ICD-9-CM Diagnosis Codes for Venous Thromboembolism

| **Conditions** | **ICD-9-CM Diagnosis Codes** |
| --- | --- |
| Pulmonary embolism | 415.1, 415.11, 415.13, 415.19 |
| Deep vein thrombosis | 451.1, 451.2, 451.81, 453.2, 453.4x, 451.83, 451.84, 451.89, 453.72, 453.73, 453.74, 453.75, 453.76, 453.77, 453.82, 453.83, 453.84, 453.85, 453.86, 453.87, 451.9, 453.8, 453.89, 453.9 |
