# Supplementary material for: Survival outcomes with warfarin compared with direct oral anticoagulants in cancer-associated venous thromboembolism in the United States: A population-based cohort study
Source: PLoS Med. 2022 May 25;19(5):e1004012. doi: 10.1371/journal.pmed.1004012 (PMC9182592; doi:10.1371/journal.pmed.1004012)
Supplement: S2 Table — ICD-O-3, International Classification of Diseases for Oncology, 3rd Edition. (DOCX) [file pmed.1004012.s004.docx]

**Supplemental Table 2.** ICD-O-3 Histology Codes for Lung Cancer Subtype

| **Subtypes** | **ICD-O-3 Histology Codes** |
| --- | --- |
| Non-small-cell lung cancer | 8010, 8012, 8013, 8020, 8046, 8050–8052, 8070–8078, 8140, 8141, 8143, 8147, 8250–8255, 8260, 8310, 8430, 8480, 8481, 8490, 8560, and 8570–8575 |
| Small-cell lung cancer | 8002, 8041, 8042, 8043, 8044, and 8045. |
