# Supplementary material for: Survival outcomes with warfarin compared with direct oral anticoagulants in cancer-associated venous thromboembolism in the United States: A population-based cohort study
Source: PLoS Med. 2022 May 25;19(5):e1004012. doi: 10.1371/journal.pmed.1004012 (PMC9182592; doi:10.1371/journal.pmed.1004012)
Supplement: S3 Table — (DOCX) [file pmed.1004012.s005.docx]

**Supplemental Table 3.** Pre- and Post-Matched Characteristics of Study Cohort

|  | **Pre-matching** | | | | | | |  | **Post-matching** | | | | | | |
| --- | --- | --- | --- | --- | --- | --- | --- | --- | --- | --- | --- | --- | --- | --- | --- |
|  |  |  | **Anticoagulant treatment group** | | |  |  |  |  |  | **Anticoagulant treatment group** | | |  |  |
|  | **Total** |  | **Warfarin** | **DOACs** | **LMWH** |  | ***p-value*** |  | **Total** |  | **Warfarin** | **DOACs** | **LMWH** |  | ***p-value*** |
|  | **n = 7,997 (%)** |  | **n = 3,056 (38)** | **n = 1,625 (20)** | **n = 3,316 (41)** |  |  |  | **n = 6,740 (%)** |  | **n = 2,696 (40)** | **n = 1,348 (20)** | **n = 2,696 (40)** |  |  |
| **Age at VTE diagnosis** | | | | | | | | | | | | | | | |
| < 75 | 3,913 (49) |  | 1,392 (46) | 769 (47) | 1,752 (53) |  | *< 0.001*^‡^ |  | 3,221 (48) |  | 1,248 (46) | 644 (48) | 1,329 (49) |  | *0.09*^‡^ |
| ≥ 75 | 4,084 (51) |  | 1,664 (54) | 856 (53) | 1,564 (47) |  |  |  | 3,519 (52) |  | 1,448 (54) | 704 (52) | 1,367 (51) |  |  |
| **Time from cancer diagnosis to VTE diagnosis** | | | | | | | | | | | | | | | |
| -6 to +3 months | 3,437 (43) |  | 1,191 (39) | 631 (39) | 1,615 (49) |  | *< 0.001*^‡^ |  | 2,765 (41) |  | 1,066 (40) | 537 (40) | 1,162 (43) |  | *0.02*^‡^ |
| > +3 months | 4,560 (57) |  | 1,865 (61) | 994 (61) | 1,701 (51) |  |  |  | 3,975 (59) |  | 1,630 (60) | 811 (60) | 1,534 (57) |  |  |
| **Primary cancer site** | | | | | | | | | | | | | | | |
| Gastric | 368 (5) |  | 134 (4) | 59 (4) | 175 (5) |  |  |  | 311 (5) |  | 113 (4) | 59 (4) | 139 (5) |  |  |
| Colorectal | 2,148 (27) |  | 1,020 (33) | 446 (27) | 682 (21) |  |  |  | 1,993 (30) |  | 930 (34) | 446 (33) | 617 (23) |  |  |
| Pancreatic | 1,189 (15) |  | 332 (11) | 207 (13) | 650 (20) |  | *< 0.001*^‡^ |  | 1,011 (15) |  | 325 (12) | 207 (15) | 479 (18) |  | *< 0.001*^‡^ |
| Lung | 3,420 (43) |  | 1,287 (42) | 742 (46) | 1,391 (42) |  |  |  | 2,840 (42) |  | 1,066 (40) | 558 (41) | 1,216 (45) |  |  |
| Ovarian | 589 (7) |  | 185 (6) | 124 (8) | 280 (8) |  |  |  | 351 (5) |  | 168 (6) | 31 (2) | 152 (6) |  |  |
| Brain | 283 (4) |  | 98 (3) | 47 (3) | 138 (4) |  |  |  | 234 (3) |  | 94 (3) | 47 (3) | 93 (3) |  |  |
| **AJCC stage** | | | | | | | | | | | | | | | |
| 0-2 | 2,416 (30) |  | 1,096 (36) | 539 (33) | 781 (24) |  |  |  | 1,950 (29) |  | 780 (29) | 390 (29) | 780 (29) |  |  |
| 3 | 1,912 (24) |  | 745 (24) | 394 (24) | 773 (23) |  |  |  | 1,860 (28) |  | 744 (28) | 372 (28) | 744 (28) |  |  |
| 4 | 2,905 (36) |  | 914 (30) | 551 (34) | 1,440 (43) |  | *< 0.001*^‡^ |  | 2,285 (34) |  | 914 (34) | 457 (34) | 914 (34) |  | *> 0.99*^‡^ |
| N/A | 411 (5) |  | 159 (5) | 63 (4) | 189 (6) |  |  |  | 315 (5) |  | 126 (5) | 63 (5) | 126 (5) |  |  |
| Unknown | 353 (4) |  | 142 (5) | 78 (5) | 133 (4) |  |  |  | 330 (5) |  | 132 (5) | 66 (5) | 132 (5) |  |  |
| **Year of VTE** | | | | | | | | | | | | | | | |
| 2012 | 1,783 (22) |  | 994 (33) | 22 (1) | 767 (23) |  |  |  | 1,534 (23) |  | 883 (33) | 21 (2) | 630 (23) |  |  |
| 2013 | 2,143 (27) |  | 866 (28) | 357 (22) | 920 (28) |  | *< 0.001*^§^ |  | 1,809 (27) |  | 767 (28) | 298 (22) | 744 (28) |  | *< 0.001*^§^ |
| 2014 | 2,290 (29) |  | 724 (24) | 652 (40) | 914 (28) |  |  |  | 1,931 (29) |  | 634 (24) | 552 (41) | 745 (28) |  |  |
| 2015 | 1,781 (22) |  | 472 (15) | 594 (37) | 715 (22) |  |  |  | 1,466 (22) |  | 412 (15) | 477 (35) | 577 (21) |  |  |
| ^‡^Fisher's exact test, ^§^Test for trend in proportions | | | | | | | | | | | | | | | |
