# Supplementary material for: Survival outcomes with warfarin compared with direct oral anticoagulants in cancer-associated venous thromboembolism in the United States: A population-based cohort study
Source: PLoS Med. 2022 May 25;19(5):e1004012. doi: 10.1371/journal.pmed.1004012 (PMC9182592; doi:10.1371/journal.pmed.1004012)
Supplement: S4 Table — (DOCX) [file pmed.1004012.s006.docx]

**Supplemental Table 4.** Socio-economic Characteristics of Study Cohort

| **Characteristics** | **Total**  **(N = 4,044)** | **Warfarin**  **(N = 2,696)** | **DOACs**  **(N = 1,348)** |
| --- | --- | --- | --- |
| Marital Status — no. (%)  Married or partnered  Unmarried(single/separated/divorced/widowed)  Unknown | 2,099 (52)  1,731 (43)  214 (5) | 1,378 (51)  1,173 (44)  145 (5) | 721 (53)  558 (41)  69 (5) |
| Median household income — no. (%)  1^st^ quarter  2^nd^ quarter  3^rd^ quarter  4^th^ quarter  Unknown | 981 (24)  913 (23)  942 (23)  866 (21)  342 (9) | 676 (25)  624 (23)  612 (23)  550 (20)  234 (9) | 305 (23)  289 (21)  330 (24)  316 (23)  108 (8) |
| Living below poverty line — no. (%)  <10%  ≥10%  Unknown | 1,985 (49)  1,717 (42)  342 (8) | 1,283 (48)  1,179 (44)  234 (9) | 702 (52)  538 (40)  108 (8) |
| Adults (≥25y) with less than high school (<12 yr) education — no. (%)  <33%  ≥33%  Unknown | 3,279 (81)  423 (11)  342 (8) | 2,172 (81)  290 (11)  234 (8) | 1,107 (82)  133 (10)  108 (8) |
